# A highly adhesive and melatonin-loaded PEG hydrogel prevents tumor recurrence and promotes wound healing for tumor-resection wound management of liposarcoma

**DOI:** 10.1016/j.mtbio.2025.101842

**Published:** 2025-05-06

**Authors:** Yonghui Liang, Jianyang Shan, Chen Tan, Zhen Pan, Zhaohui Li, Xiang Fei, Gen Wen, Qingcheng Yang, Dongdong Cheng

**Affiliations:** a Department of Orthopedic Surgery, Shanghai Sixth People's Hospital Affiliated to Shanghai Jiao Tong University School of Medicine, Shanghai, 200233, China; b Institute of Clinical Chemistry and Laboratory Medicine, Section Mass Spectrometry and Proteomics, University Medical Center Hamburg-Eppendorf (UKE), Hamburg, 20246, Germany

**Keywords:** Liposarcoma, Tumor recurrence, PEG hydrogel, Melatonin, Wound healing

## Abstract

Liposarcoma is the most common soft tissue sarcoma, and the surgical resection is the primary treatment. However, liposarcoma recurrence and delayed wound healing remain critical challenges due to extensive surgical interventions. Herein, we design an injectable polyethylene glycol (PEG)-based hydrogel (PEG@MT) with high skin adhesion and loading melatonin, to concurrently prevent liposarcoma recurrence and promote postoperative wound healing. The hydrogel is formed by mixing 8-arm-polyethylene glycol-succinimidyl glutarate (8-arm-PEG-SG) and 4-arm-polyethylene glycol-amine (4-arm-PEG-NH_2_) at a 1:1 ratio, establishing an amide-bond crosslinked network. Excess succinimidyl ester groups bind to endogenous skin amine groups, ensuring high adhesion for effective wound closure. Functional assays demonstrated its dual functionality: promoting wound healing and angiogenesis while suppressing liposarcoma proliferation and metastasis. Mechanistically, the PEG@MT hydrogel promotes cell cycle arrest and apoptosis via FOXO pathway in liposarcoma cells. The studies also established a novel liposarcoma resection model to validate therapeutic outcomes. The PEG@MT hydrogel offers effective closure of the wound due to the high skin adhesion capacity. Meanwhile, the sustained release of melatonin in surgical wounds significantly inhibits the local recurrence of liposarcoma. Overall, the developed PEG@MT hydrogel capable of preventing recurrence of liposarcoma while promoting postoperative wound healing shows valuable application for postsurgical liposarcoma-resection wound management.

## 1 Introduction

Liposarcoma is the most common soft tissue sarcoma [1], and nearly 50 % of liposarcoma is localized in the deep, subfascial soft tissues of the thighs [2]. The limb-sparing surgical resection and the local radiotherapy are the main therapies for liposarcoma [3,4]. However, there are two main problems in liposarcoma therapy. Firstly, nearly 20 % patients would face the local recurrence despite the standard therapy, impacting on the overall survival rate [5]. Meanwhile, postoperative wound healing in the liposarcoma is another important clinical problem due to a large tumor volume and adverse effects of radiotherapy. A large tumor would cause a wide and irregular range of skin wounds, causing a high tension when suturing, in turn, causing series of complications such as dehiscence and infection [6]. In addition, the local radiotherapy has been reported to have high complications such as late toxicity to the skin and wound healing problem in liposarcoma [7]. Thus, it is necessary to explore a new effective way to solve both tumor recurrence and wound healing in the liposarcoma.

Based on the problems of postsurgical wound healing in tumors many studies focused on the application of hydrogels, especially in the melanoma [8,9]. However, the high skin tension of the large, irregular wound often exists in liposarcoma, limiting the application of many types of hydrogels, and thus, it has not been reported about the application of hydrogel in the postsurgical wound in liposarcoma. In order to reduce the complications of conventional sutures promote wound closure and healing, and ensure the convenient application in surgery in liposarcoma, the hydrogels with characteristic of high wet adhesion, high strength, and injectable capacity should be developed [10,11]. Based on these problems, we believed the polyethylene glycol (PEG)-based hydrogel could be a good choice. Firstly, the PGE is the Food and Drug Administration (FDA)-approved material because of its good biocompatibility [12]. Secondly, the PEG-based hydrogels can carry different reactive groups to form covalent linkages, including amide bonds [13] and carbon-sulfur bonds [14], providing a high adhesion. In addition, the PEG-based hydrogels are injectable because the PEG precursors can premix, inject as a liquid, and finally solidified and anneal with the wound environment [15], through different reactions [16,17]. In addition, the PEG-based hydrogel also has the function of local drug delivery. The drug can be simply mixed into the hydrogel precursors before injection, and the sustained release of drug is accompanied by hydrogel degradation [15]. Combined with the advantages of PEG, it will be valuable for the application of PEG-based hydrogel to address postoperative wound repair challenges in liposarcoma.

Considering the drugs loaded in hydrogel, the melatonin was taken into our consideration to address the problems of tumor recurrence and wound healing simultaneously in liposarcoma. Melatonin (MT), N-acetyl-5-methoxytryptamine, is a natural indole compound that is synthesized and secreted primarily by the pineal during sleep. Recently, the multiple functions of melatonin have been studied including tumor inhibition [18] and wound healing promotion [19]. The melatonin is a valuable anti-cancer agent which can inhibit tumor progression, metastasis, and recurrence in many kinds of cancer [20,21]. A randomized controlled trial of lung cancer with 709 patients showed the melatonin can increase the Disease-Free Survival (DFS) in patients with late-stage disease, demonstrating its ability of tumor recurrence inhibition [22]. Mechanistically, the melatonin can arrest cell cycle, promote cellular apoptosis, and regulate many signaling pathways to inhibit the tumor progression [23]. However, the anti-cancer capacity of melatonin to liposarcoma has not been reported. In addition, the studies of melatonin have extended to the wound healing area, due to its excellent anti-inflammatory and antioxidant capacity [19]. The melatonin can also promote the cell viability of fibroblasts and angiogenesis in the wound environment [24]. Based on the multifunction in tumor inhibition and wound healing promotion, we choose the melatonin as the drug loading in the hydrogel for a local tumor-wound niche in liposarcoma.

**Fig. 1:**
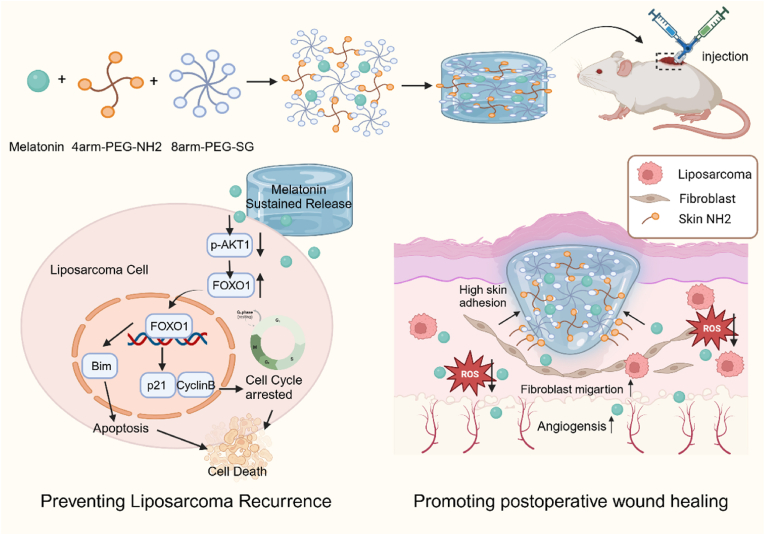
An injectable hydrogel with high adhesion property and melatonin loaded for efficiently preventing tumor recurrence and simultaneously promoting wound healing during the postsurgical tumor treatment in liposarcoma. This figure was created in BioRender. Liang, Y. (2025) https://BioRender.com/372eusg.

In this study, we develop a melatonin-loaded injectable PEG hydrogel (PEG@MT) with high skin adhesion to prevent tumor recurrence and promote wound healing after liposarcoma resection (Fig. 1). The injectable PEG hydrogel was formed by mixing 8-arm-PEG-SG and 4-arm-PEG-NH_2_ with the 1:1 M ratio under the mild conditions. The mixture of 1:1 M ratio facilitates an additional connection between the succinimidyl ester groups of 8-arm-PEG-SG and the amino groups in wound skin, providing a higher skin adhesion. Regarding the biological function, the PEG@MT hydrogel developed an excellent biocompatibility both in vitro and in vivo, and showed the ability of wound healing through regulating fibroblast proliferation, angiogenesis, and antioxidant activity. We also demonstrated the anticancer effects of the PEG@MT hydrogel in vitro including tumor proliferation and metastasis inhibition in liposarcoma. Mechanistically, the PEG@MT hydrogel enhances FOXO signaling pathway to promote cell cycle arrest and apoptosis in liposarcoma cells. Finally, we developed a novel liposarcoma resection mice model, and the PEG@MT hydrogel exhibited an outstanding ability of tumor recurrence inhibition and wound healing promotion in vivo. Overall, our studies demonstrated the PEG@MT hydrogel as a versatile hydrogel in regulation of tumor recurrence and wound healing, hence pinpointing a great potential in the postoperative treatment of liposarcoma.

## 2 Result and discussion

### 2.1 Characterization of PEG@MT hydrogel

**Fig. 2:**
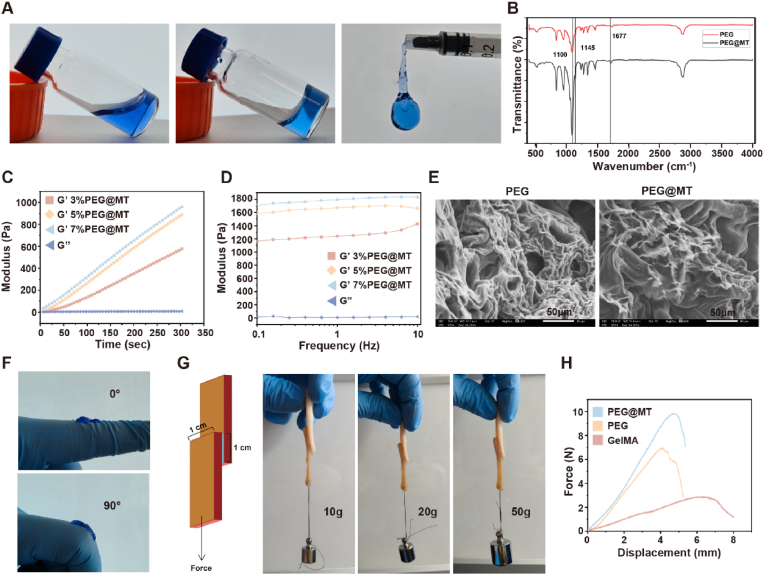
**Preparation and characterization of PEG@MT hydrogel.** (A) Photographs of PEG@MT hydrogel before and after gelation, and the injectable property of PEG@MT hydrogels. (B) FT-IR spectrum of PEG hydrogel and PEG@MT hydrogel. (C) Rheological time scanning plots of PEG@MT hydrogels with different solid content. (D) Oscillatory frequency sweeps of PEG@MT hydrogels with different solid content. (E) SEM images of PEG hydrogel and PEG@MT hydrogel. (F) PEG@MT hydrogel (Trypan Blue-stained) at different curvatures of the hand joints. (G) Adhesion properties of the PEG@MT hydrogel on pig skin; (H) Adhesion strength between PEG hydrogel, PEG@MT hydrogel and pig skin. (For interpretation of the references to colour in this figure legend, the reader is referred to the Web version of this article.)

A PEG hydrogel based on 8-arm-PEG-SG and 4-arm-PEG-NH_2_ was produced by simply mixing their solutions (ratio of 1:1). The amino groups of 4-arm-PEG-NH_2_ and the succinimide group of 8-arm-PEG-SG undergo rapid amidation-mediated crosslinking to form a hydrogel. And the PEG@MT hydrogel was produced under the MT doping condition. The structure of 8-arm-PEG-SG and its NMR spectra was shown (Fig. S1). A vial turnover test demonstrated the PEG@MT hydrogel was successfully prepared. Besides, the process from liquid mixture to hydrogel formation was shown and demonstrated the injectable function of the PEG@MT hydrogel (Fig. 2A). To test the cross-linking of hydrogels, the FT-IR was conducted. According to FT-IR spectra of PEG, the results showed that significant antisymmetric stretching vibration peaks of the C-O-C ether bonds were observed at 1100 cm^−1^ and 1145 cm^−1^, indicating that the PEG backbone structure remained stable during the cross-linking process [25,26]. Compared to PEG, PEG@MT exhibited a slight red shift in this spectral region, accompanied by a slight decrease in absorption peak intensity. Then, the strong absorption peak at 1677 cm^−1^ was attributed to the C=O stretching vibration of the amide I band, confirming that the amino groups (-NH_2_) from 4-arm-PEG-NH_2_ reacted with the succinimidyl ester groups of 8-arm-PEG-SG via amidation reactions, forming a covalent cross-linked network [27]. Besides, at 1650 cm^−1^, a distinct absorption peak emerged in PEG@MT, which is ascribed to the amide C=O stretching vibration of the melatonin molecule [28] (Fig. 2B). This observation confirms the presence and successful incorporation of melatonin within the PEG matrix. In conclusion, the FT-IR results validated the covalent cross-linking mechanism, constructing a stable melatonin-loaded PEG hydrogel system. In addition, in the rheological characterization of PEG@MT hydrogel formation, time-dependent modulus measurements were performed on hydrogels with 3 %, 5 %, and 7 % solid content. The slope of the G′ versus time increased progressively with higher solid content (3 % < 5 % < 7 %), indicating faster network formation and enhanced cross-linking density at elevated polymer concentrations. The G″ remained near 0 Pa throughout the gelation process, confirming the dominance of elastic properties (G' ≫ G″) (Fig. 2C). Frequency sweeps rheological tests revealed that G' ≫ G'' (resembling a purely elastic solid) aligns with time-sweep data, further validating the stability of the covalent network [29]. It also can be observed that G′ increased significantly with solid content, but the growth rate slowed between 5 % and 7 %, suggesting potential saturation of cross-linking efficiency at higher concentrations (Fig. 2D). Thus, we chose 5 % (w/v) as the suitable solid content for further experiments and application. Besides, the Scanning electron microscopy (SEM) characterization revealed a three-dimensional porous network with pore sizes ranging from 20 to 30 μm in both the PEG and PEG@MT (Fig. 2E) [30]. Our studies also showed the time-dependent degradation of PEG@MT hydrogel in vitro, and its weight lose nearly 75 % within 10 days (Fig. S2A). In addition, the melatonin loading and release in PEG@MT hydrogel were explored, and the Drug Loading Content (DLC%) and the Encapsulation Efficiency (EE%) were (0.81 ± 0.04)% and (81.4 ± 4.32)%, respectively (Fig. S2B). In the drug release experiment, the results showed that the release rate of melatonin in the PEG@MT hydrogel exceeded 50 % within 2 days, and released nearly 90 % within 5 days (Fig. S2C).

In order to evaluate the adhesion efficacy of the PEG@MT hydrogel for wounds, assessments of its adhesion property on the finger joint were conducted. The PEG@MT hydrogel, which was dyed blue for enhanced visibility, was applied to the finger joint and subsequently subjected to the ranges of 0 and 90°. The hydrogel remained firmly adhered throughout the motions (Fig. 2F). This result highlights the PEG@MT hydrogel's strong adherence capabilities to the wound, even under the stress of joint movement. Furthermore, to further quantitatively investigate the skin adhesion efficacy of the PEG@MT hydrogel, a pig skin adhesion test was conducted. 1 cm^2^ hydrogel was used to adhere two pig skins and the different weights (10 g, 20 g and 50 g) were linked to one side. The results showed a strong and stable adhesion of the PEG@MT hydrogel toward the pig skin under the pull of 10g, 20g and 50g weights (Fig. 2G). In the displacement-force curves, we used the GelMA hydrogel as a control group, and the fracture occurred at 2 N with a displacement of 6 mm; however, in PEG hydrogel, the fracture occurred at nearly 7 N with a corresponding displacement of 4 mm, demonstrating significantly higher adhesion compared to GelMA hydrogel. Besides, the slopes of both PEG and PEG@MT hydrogels were similar, indicating that melatonin incorporation did not compromise adhesive strength (Fig. 2H). Overall, pig skin adhesion tests demonstrated that PEG and PEG@MT hydrogels exhibit superior adhesive strength to the skin tissue and a potential value of clinical application. The PEG-based hydrogel is formulated via a 1:1 stoichiometric reaction between 8-arm-PEG-SG and 4-arm-PEG-NH_2_, forming an amide-bonded network. Additionally, the excess succinimidyl ester (-SG) groups in 8-arm-PEG-SG enable covalent binding to endogenous amine groups in skin tissues, amplifying interfacial adhesion [31]. This dual mechanism of intrinsic network stabilization and reactive tissue anchoring ensures strong adhesion even under dynamic physiological conditions. Thus, we believe the PEG-based hydrogel can solve the problems of large, irregular wounds with high tension after an extensive surgical resection, and avoid the complication of conventional sutures, including wound dehiscence and infection [32] for postoperative wound management in liposarcoma.

### 2.2 Biocompatibility and Wound Healing study

**Fig. 3:**
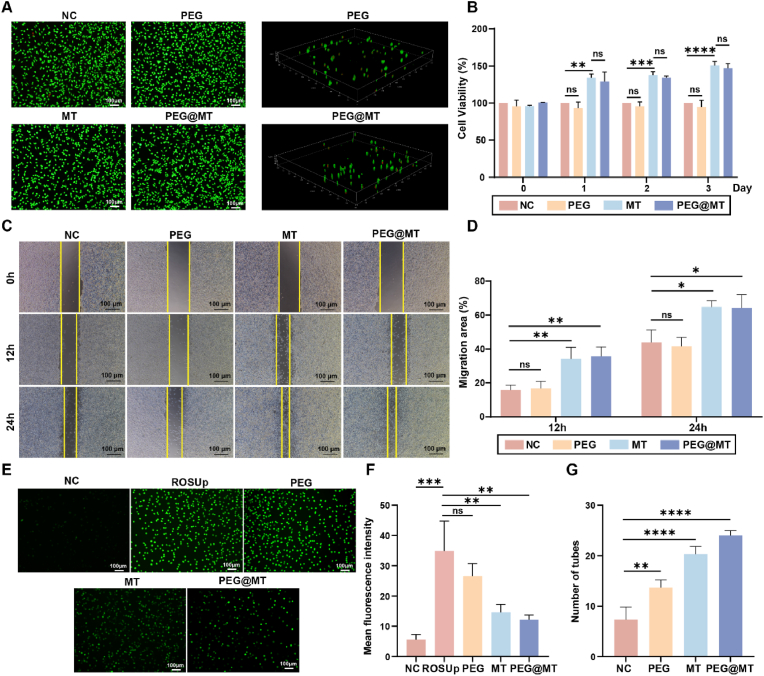
**Biocompatibility and Wound Healing effect of PEG@MT hydrogel.** (A) Live/dead staining of L929 cells after incubation with the PEG hydrogel, melatonin, and PEG@MT hydrogel for 48 h (n = 3). (B) Effects of PEG hydrogel, melatonin and PEG@MT hydrogel on the activity of L929 cells after the treatment of 1 day, 2 days and 3 days (n = 3). (C) Cell migration effect of L929 cells after incubation with PEG hydrogel, melatonin and PEG@MT hydrogel (n = 3). (D) The quantitative analysis of the L929 cell scratch experiment. (E) The ROS level of L929 cells after incubation with the PEG hydrogel, melatonin, and PEG@MT hydrogel for 48 h (n = 3). (F) The quantitative analysis of ROS level in L929 cells. (G) The angiogenesis of HUVEC cells after incubation with the PEG hydrogel, melatonin, and PEG@MT hydrogel for 48 h (n = 3). ∗p < 0.05; ∗∗p < 0.01; ∗∗∗p < 0.001; ∗∗∗∗p < 0.0001.

Biocompatibility is a crucial for biomedical applications. Therefore, we investigated the cytotoxicity of the PEG@MT hydrogel using the Live/dead staining in vitro. The cytotoxicity of the PEG and the PEG@MT hydrogel were assayed by co-culturing fibroblast cells in media with the hydrogels. The results show that both the PEG and PEG@MT hydrogel displayed low toxicity to L929 mouse fibroblast cells for 48 h treatment (Fig. 3A). In addition, to further explore the biocompatibility of PEG@MT hydrogel in vivo, the PEG@MT hydrogel was embedded subcutaneously in 6-weeks-old rats, and the results demonstrated that the PEG@MT hydrogel gradually degraded within 7 days (Fig. S3), while the rats embedded with the PEG@MT did not demonstrate significant visceral toxicity of skin or other organs compared to the control-group rats according to the HE staining (Fig. S4), as well as the inflammation in vivo according to the TNF-α in serum of rats (Fig. S5). Overall, these findings indicate that PEG@MT hydrogel exhibits excellent biocompatibility and degradation, and is promising to be biocompatible wound dressing and implantable materials in postoperative treatment of liposarcoma.

After surgical removal of large liposarcomas, postoperative wound healing is very difficult, so promoting rapid proliferation and migration of fibroblasts, decreasing ROS level and promoting angiogenesis is essential for wound repair [33]. Therefore, we investigated the function of PEG@MT in wound repair through a series of experiments in vitro. In the CCK8 assay, compared to NC group, both the MT group and the PEG@MT group exhibited a significant cell viability of L929 cells with 1, 2, 3 days treatment (Fig. 3B). In the cell scratch experiment, the MT and the PEG@MT significantly enhanced the migration ability of L929 cells with 12 h and 24 h treatment (Fig. 3C). And quantitative analysis indicated that the migration ratio of PEG@MT group (64.2 %) was significantly higher than the control group (43.9 %) after 24 h treatment, confirming the PEG@MT could effectively accelerate the migration of fibroblasts (Fig. 3D). In addition, the level of ROS will be enhanced in the postoperative wound, and the high ROS levels usually cause local irreversible oxidative damage, leading to the elevated inflammation. It is demonstrated that decreasing the level of ROS is pivotal to the healthy functioning of fibroblasts, promoting healing in the postoperative wound [34]. Thus, we tested the ROS level in the H_2_O_2_ pre-treated L929 cells after MT and PEG@MT treatment. The results showed that MT and the PEG@MT can effectively decrease the ROS induced by H_2_O_2_ in L929 cells, demonstrating the strong anti-oxygen ability of the PEG@MT hydrogel (Fig. 3E and F). Besides, the angiogenesis plays an important role in wound repair. The new blood vessels support cells with nutrition and oxygen through blood supply, and transport grow factors to promote wound healing [35]. In the tube formation assay, compared to NC group, the number of tubes were significantly higher in the MT and PEG@MT group, exhibiting the ability of promoting angiogenesis in the two group (Fig. 3G; Fig. S6). Overall, all these experiments demonstrated that PEG@MT can promote the cell viability and migration ability of fibroblast, decrease the ROS levels and promote angiogenesis, creating a pro-repair environment in the postoperative wound.

### 2.3 In Vitro Anticancer Efficacy of PEG@MT hydrogel

**Fig. 4:**
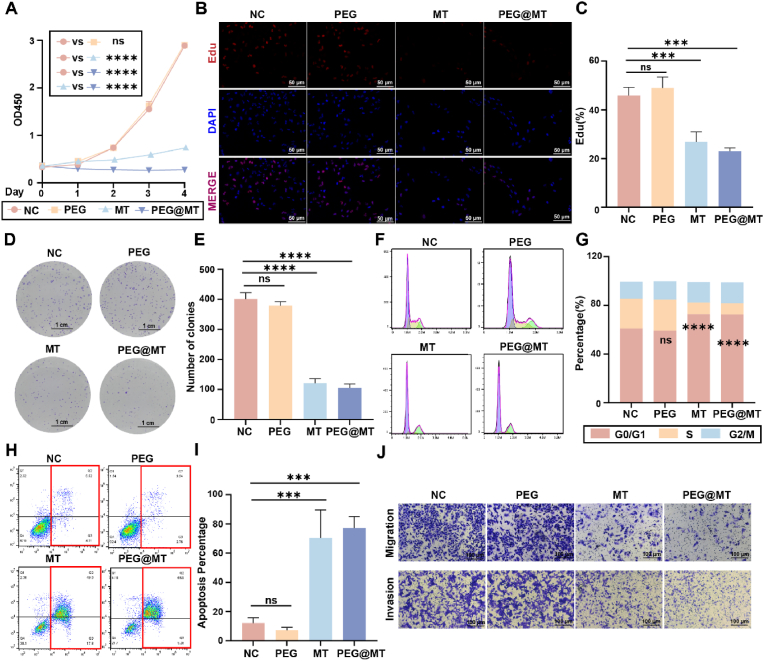
**In Vitro Anticancer Efficacy of PEG@MT Hydrogel.** (A) The cells viability of SW872 cells after incubation with the PEG hydrogel, melatonin, and PEG@MT hydrogel for different time (n = 3). (B–C) The Edu assay and the ratio of Edu positive SW872 cells after incubation with the PEG hydrogel, melatonin, and PEG@MT hydrogel for 48 h (n = 3). (D–E) The clone formation assay of SW872 cells with the treatment in (B) (n = 3). (F–G) The cell cycle analysis with the treatment in (B) in SW872 cells (n = 3). (H–I) The Annexin V-PI staining to test cellular apoptosis with the treatment in (B) in SW872 cells (n = 3). (J) The transwell assay to test the migration and invasion capacity in SW872 cells with the treatment in (B) (n = 3). ∗∗p < 0.01; ∗∗∗p < 0.001; ∗∗∗∗p < 0.0001.

To test the ability of preventing tumor recurrence, the in vitro anticancer efficacy of PEG@MT hydrogel against the liposarcoma cell line SW872 was systemically studied. We first examined the cell viability of SW872 cells after different treatment for 4 days, and the results showed that MT and PEG@MT hydrogel groups can inhibit the proliferation of SW872 cells after 4 days of treatment, while the PEG group exhibited the minimal inhibition (Fig. 4A). Importantly, the inhibitory effect was significantly more apparent in the PEG@MT hydrogel group compared to the MT group after 4 days of treatment, demonstrating the sustainable release capability of the PEG@MT hydrogel. To further explore the anticancer efficacy, the Edu assay and the colony formation assay were performed. The SW872 cells were incubated with the PEG or PEG@MT hydrogel, or treated with MT for 48 h, followed by cell harvesting for the assays. In the Edu assay, the Edu-positive ratios were 26.8 % and 23 % in the MT and PEG@MT hydrogel group, respectively, which were lower than 45.9 % in the control group (Fig. 4B and C). Additionally, the results of the colony formation assay showed reduced colony numbers in both the MT and PEG@MT hydrogel groups compared to the control group (Fig. 4D and E). All these results demonstrated that the MT and PEG@MT hydrogel effectively inhibit the proliferation of liposarcoma cells, with the PEG@MT hydrogel showing sustained inhibition due to its controlled release function.

To investigate the mechanism of PEG@MT-mediated proliferation inhibition, we conducted further experiments to explore its effect on cell cycle and apoptosis of SW872 cells. We conducted propidium iodide (PI) DNA staining by flow cytometry to analyze the cell cycle. The PEG group showed the minimal impact, whereas both MT and PEG@MT hydrogel groups significantly arrested SW872 cells at G0/G1 phase compared to control group (Fig. 4F). Quantitative analysis revealed a G0/G1 phase cell ratio of 72.5 % in the PEG@MT hydrogel group, higher than the 61.0 % in the control group. Conversely, the S phase cell ratio decreased to 8.9 % in the PEG@MT hydrogel group compared to 24.3 % in the control group (Fig. 4G), indicating reduced actively proliferating cells. These results suggest G1 checkpoint arrest under PEG@MT hydrogel treatment in liposarcoma cells. In addition, Annexin V/PI staining by flow cytometry showed apoptosis rates of 70 % and 77 % in the MT and PEG@MT hydrogel groups, respectively, significantly higher than the 12 % in the control group, demonstrating the PEG@MT hydrogel effectively promotes apoptosis of liposarcoma cells (Fig. 4H and I). Furthermore, MT and PEG@MT hydrogel effectively inhibited SW872 cell migration and invasion, demonstrating anti-metastatic potential (Fig. 4J; Fig. 7, S8 and S9).

In the molecular spectrum of liposarcoma, CDK4, the key regulator of the G1 checkpoint, is amplified in the undifferentiated subtype with high recurrence probability [36]. Our findings demonstrate that PEG@MT hydrogel induces G1 cell cycle arrest, promotes apoptosis, and inhibits liposarcoma cell proliferation/metastasis, highlighting its targeting value and superior anticancer efficacy in liposarcoma.

### 2.4 The RNA-sequencing analysis of PEG@MT Hydrogel and Validation Experiment

**Fig. 5:**
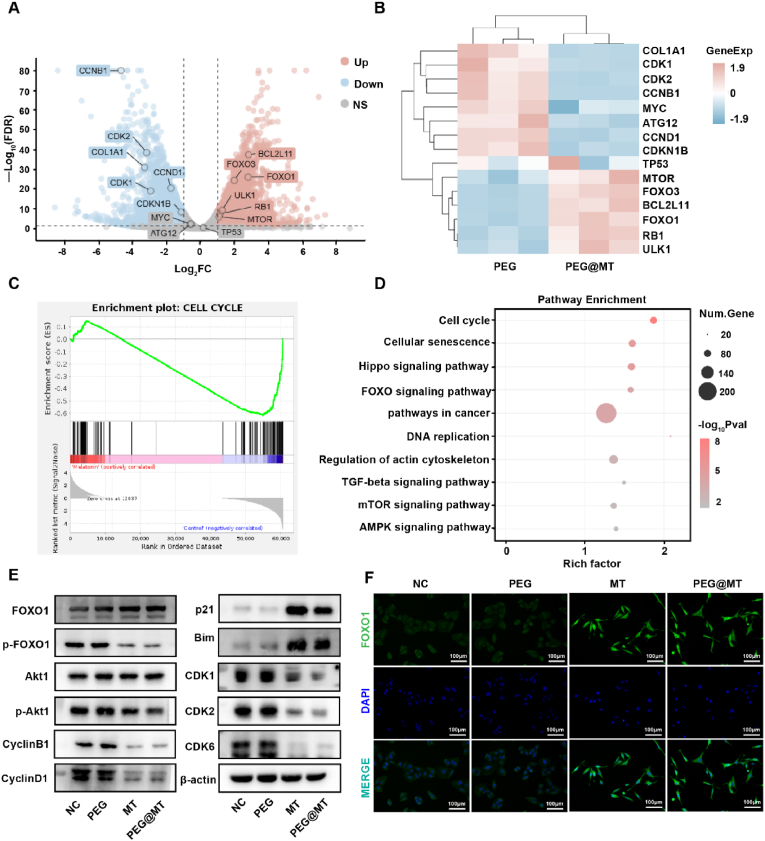
**The RNA-Sequencing Analysis of PEG@MT Hydrogel and Validation Experiment.** (A) The Volcano plots displayed the differentially expressed genes between SW872 cells with PEG hydrogel and PEG@MT hydrogel. (B) Heat-map of gene expression in SW872 cells treated with PEG hydrogel and PEG@MT hydrogel. (C) GSEA analysis showed the genes set of cell cycle in SW872 cells treated with PEG hydrogel and PEG@MT hydrogel. (D) The enriched pathways were shown by the Kyoto Encyclopedia of Genes and Genomes (KEGG) analysis in SW872 cells with PEG hydrogel and PEG@MT hydrogel treatment. (E) The western blot was used to detect the marker in FOXO signaling pathway and the cell cycle signaling pathway in SW872 cells (n = 3). (F) The immunofluorescent staining in SW872 cells to detect the expression and the nuclear localization of FOXO1 (n = 3). The RNA-Sequencing Analysis included three replicates per group in (A–D).

As we initially explored the anticancer mechanisms of PEG@MT hydrogel, we further conducted RNA-sequencing analysis on SW872 cells treated with PEG hydrogel or PEG@MT hydrogel. Notably, 2370 genes were up-regulated, and 2005 genes were down-regulated in cells treated with PEG@MT hydrogel compared to those treated with PEG hydrogel in the volcano plot analysis (Fig. 5A), with representative gene symbols shown in the heatmap (Fig. 5B). Furthermore, both Gene Set Enrichment Analysis (GESA) and Kyoto Encyclopedia of Genes and Genomes (KEGG) Enrichment Analysis showed that the cell cycle pathway was significantly down-regulated under PEG@MT hydrogel treatment, supporting our previous cell cycle experiment conclusions (Fig. 5C and D). In the KEGG analysis, the PEG@MT hydrogel predominantly suppressed gene expression in the Hippo signaling pathway and enhanced gene expression in the FOXO signaling pathway (Fig. 5D). The Hippo signaling pathway is up-regulated in many cancers, where YAP/TAZ act as the key factor to promote tumor progression, especially metastasis [37]. Other study has demonstrated that MT can inhibit the YAP expression and Hippo pathway in hepatocellular carcinoma [38], consistent with our PEG@MT hydrogel results in liposarcoma cells. Surprisingly, we observed activation of the FOXO signaling pathway, which has not been previously reported in studies of melatonin. FOXOs are considered tumor suppressors due to their role in promoting cell cycle arrest and apoptosis [39], aligning with our previous experiments. Thus, we hypothesis the PEG@MT hydrogel activates the FOXO signaling pathway to inhibit tumor proliferation and recurrence in liposarcoma.

To validate the RNA-sequencing results, we conducted Western Blot and Immunofluorescence assay to analyze key proteins in the FOXO signaling pathway and cell cycle pathway. In the FOXO pathway, FOXO1 is phosphorylated by phosphorylated Akt1 and translocated from nucleus to cytoplasm. When the FOXO pathway is activated, the p-FOXO is dephosphorylated and the FOXO1 enters the nucleus and regulates the gene promoter downstream [40]. The Western Blot results showed the increased FOXO1 expression and the decreased phosphorylated FOXO1 in the MT and PEG@MT hydrogel group (Fig. 5E; Fig. S10A and B). Upstream of FOXO1, PEG@MT hydrogel inhibited the p-Akt1(Fig. 5E; Fig. S10C and D). Immunofluorescence assay and Western Blot Assay for nuclear protein confirmed enhanced nuclear FOXO1 expression and the nuclear localization in MT and PEG@MT hydrogels (Fig. 5F; Fig. S11A–C), indicating FOXO pathway activation. Additionally, MT and PEG@MT hydrogel inhibited the expression of cyclinB1, cyclinD1, CDK1, CDK2 and CDK6 while upregulating p21(Fig. 5E; Fig. S10E-G, I-K). All these results showed the cell cycle was arrested by the MT and PEG@MT hydrogel treatment in liposarcoma cells. MT and PEG@MT hydrogel also promoted the expression of pro-apoptosis protein Bim (Fig. 5E; Fig. S10H). The cyclinB1, cyclinD1, p21 and Bim proteins are all the key factors regulated by FOXO1 [41]. Our results demonstrated the MT and PEG@MT hydrogel induced the cell arrest and apoptosis through activating FOXO1 and its subsequently regulation of the downstream factor including cyclinB1, cyclinD1, p21 and Bim proteins.

### 2.5 In vivo liposarcoma recurrence inhibition and wound healing promotion in a liposarcoma resection model

**Fig. 6:**
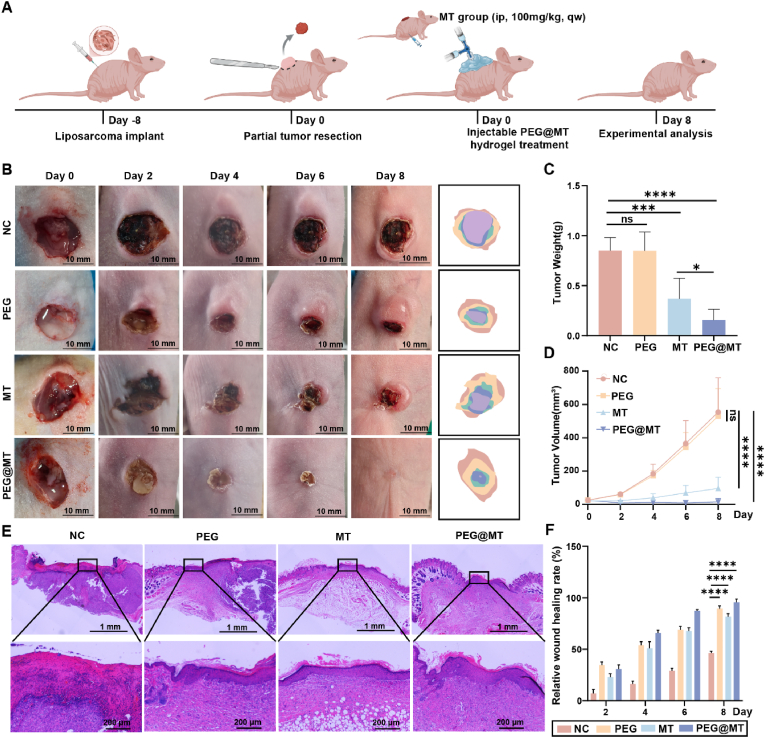
**In vivo tumor recurrence inhibition and wound healing promotion in a liposarcoma resection model.** (A) The liposarcoma resection model was used to the test the capacity of PEG@MT hydrogel in tumor recurrence inhibition and wound healing promotion. This figure was created in BioRender. Liang, Y. (2025) https://BioRender.com/smogfhj. (B) Photographs of tumor/wound sites in different groups during the 8-day treatment period. (C) The tumor weight after the treatment of PEG hydrogel, melatonin, and PEG@MT hydrogel for 8 days (n = 6). (D) The tumor weight after the treatment of PEG hydrogel, melatonin, and PEG@MT hydrogel at different time (n = 6). (E) The HE analysis of the wounds in different groups on day 8. (F) The quantitative analysis of the wound healing area in different groups at different time (n = 6). ∗p < 0.05; ∗∗∗p < 0.001; ∗∗∗∗p < 0.0001.

In order to explore the function of PEG@MT hydrogel on both inhibiting liposarcoma recurrence and promoting wound healing simultaneously in vivo, we developed a novel liposarcoma resection model [42]. The SW872 cells were harvested and injected subcutaneously into the 4-week-old nude mice. Then, approximately 95 %-size tumors were resected when the tumors grew to about 200 mm^3^. The MT group was conducted by intraperitoneal injection with 200 μL of a solution of melatonin (100 mg/kg, every 2 days), and in the PEG@MT group, the PEG@MT hydrogel was injected and formed a covering over the postoperative wound. Then the wound healing process and the tumor size were recorded on days 2, 4, 6 and 8 after surgery (Fig. 6A). Representative images were shown in Fig. 6B. The results showed that the size and weight of the tumor continued to enhance after resection in the PEG group and the control group. However, the tumor size and weight in the MT group decreased significantly compared to the control group, and tumors in PEG@MT hydrogel group nearly disappeared within 8 days postoperatively (Fig. 6C and D; Fig. 12). These results demonstrated that PEG@MT hydrogel can effectively inhibit the recurrence of liposarcoma in vivo. In the evaluation of wound healing, the wounds in control group hardly healed and developed obvious scabs within 8 days. However, the wounds in PEG group were significantly smaller due to its high skin adhesion, and the MT group, due to its own properties, also showed significantly wound healing promotion compared to the control group. Surprisingly, the wounds in PEG@MT hydrogel group nearly healed (with an approximately 90 % healing area) within 8 days (Fig. 6B–F). Histology analysis was conducted to further investigate the wound healing process. The results of H&E staining showed that the neo-epidermis was hardly observed, and the large blood scabs were still consistent in the control group in day 8. However, the reconstructed epitheliums formed in the PEG, MT and PEG@MT hydrogel group, and the new hair of follicles could be observed near the wound in the PEG@MT hydrogel group (Fig. 6E). Overall, these results demonstrated that the PEG@MT hydrogel group exhibited superior wound contraction, a smaller unclosed wound area, and excellent ability to inhibit tumor recurrence while promoting wound healing in liposarcoma mouse model.

**Fig. 7:**
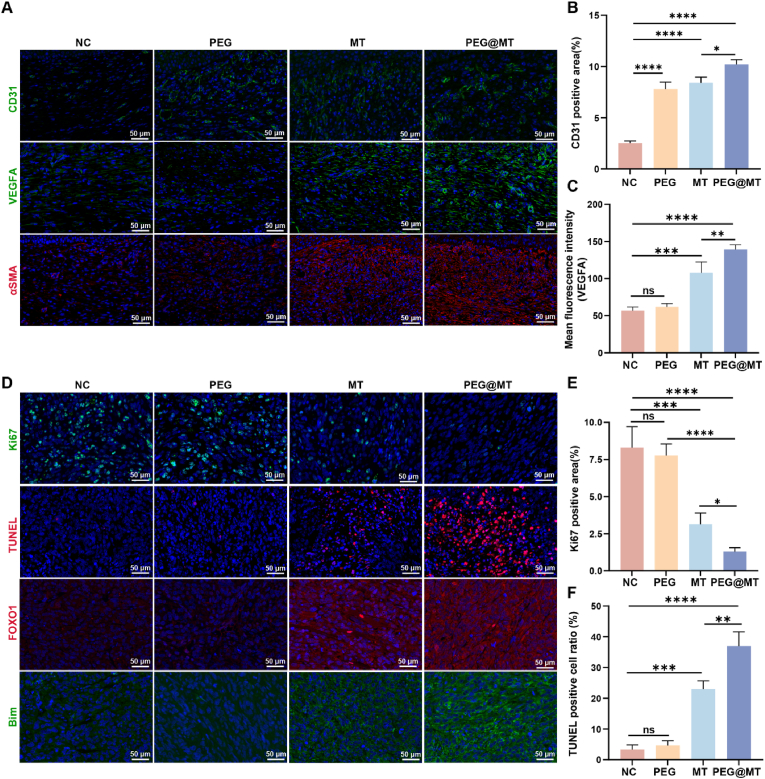
**The immunofluorescent staining of the wound skin tissue and liposarcoma tissue in vivo.** (A) The expression of CD31, VEGFA and αSMA in the wound skin tissue after the treatment of PEG hydrogel, melatonin, and PEG@MT hydrogel for 8 days (n = 3). (B) The quantitative analysis of the CD31 positive area (n = 3). (C) The quantitative analysis of the VEGFA mean fluorescence intensity (n = 3). (D) The expression of Ki67, FOXO1 and Bim protein, and the TUNEL staining in the liposarcoma tissue after the treatment of PEG hydrogel, melatonin, and PEG@MT hydrogel for 8 days (n = 3). (E) The quantitative analysis of the Ki67 positive area (n = 3). (F) The quantitative analysis of the TUNEL positive area (n = 3). ∗p < 0.05; ∗∗p < 0.01; ∗∗∗p < 0.001; ∗∗∗∗p < 0.0001.

The PEG@MT hydrogel combines the advantages of PEG-based hydrogel and melatonin. The PEG hydrogel, with amide bond formation, offers high strength and strong skin adhesion, allowing it to effectively adhere to the wound while accommodating skin movement [43]. In addition, the sustained release of melatonin in PEG@MT hydrogel is beneficial for tumor inhibition and wound repair, which is considered as a slow process [44]. Thus, we believe that PEG@MT hydrogel is suitable for postoperative wounds in liposarcoma.

### 2.6 In vivo anticancer and angiogenesis efficacy of PEG@MT hydrogel

To investigate tumor inhibition and wound healing promotion in vivo, we conducted histological immunofluorescence analysis. First, CD31, a marker of angiogenesis, and VEGFA, a key promoter in angiogenesis, were chosen for immunofluorescence analysis. The level of CD31 was increased in the PEG and MT groups compared to the control group, and the PEG@MT hydrogel group showed the highest expression of CD31 in the wound compared to the other groups (Fig. 7A and B). Similarly, the expression of VEGFA was significantly enhanced both in MT and PEG@MT group compared to the control group, and the PEG@MT hydrogel exhibited better effect than the MT group (Fig. 7A–C). In addition, αSMA, a marker of the fibroblast activation, were used to detect the migration ability of fibroblast in vivo. The results showed that both the MT and PEG@MT hydrogel can enhance the expression of αSMA, and the PEG@MT hydrogel showed the best effect compared to other groups (Fig. 7A; Fig. S13). In brief, PEG@MT hydrogel effectively promoted wound healing and angiogenesis in a tumor-resection wound. Next, tumor proliferation inhibition was measured by Ki67 staining (a proliferation marker) and the terminal deoxynucleotidyl transferase dUTP nick-end labeling (TUNEL) staining (an apoptosis marker). The results showed that PEG hydrogel did not affect tumor proliferation or apoptosis compared to the control group, but the MT group showed lower Ki67 expression, and a higher apoptosis rate compared to the control group. Surprisingly, the PEG@MT hydrogel group exhibit the lowest Ki67 expression and the highest TUNEL area compared to the other groups (Fig. 7D). The significant differences among the different groups were further verified by the quantitative analysis (Fig. 7E and F). These results demonstrated that PEG@MT hydrogel can effectively inhibit the proliferation and promote apoptosis in liposarcoma in vivo. In addition, in previous mechanistic studies, the FOXO signaling pathway was found to be enhanced during the tumor inhibition process by PEG@MT hydrogel. Thus, we conducted FOXO1 and Bim staining via immunofluorescence analysis in liposarcoma tissues. Consistently, both the expression and the nuclear localization of FOXO1 were increased in the MT and PEG@MT hydrogel groups. Additionally, Bim, an apoptosis marker activated by FOXO1, was also upregulated in the MT and PEG@MT hydrogel groups (Fig. 7D; Figs. S14 and S15). These finding demonstrate that PEG@MT hydrogel can activate the FOXO signaling pathway and promote apoptosis by upregulating Bim in the liposarcoma in vivo.

In our experiment, the PEG hydrogel alone was also able to promote the angiogenesis even without melatonin loading and enhanced VEGFA expression. The PEG-based hydrogel features a three-dimensional porous network, providing a favorable environment for angiogenesis in the wound. When combined with melatonin, the PEG@MT hydrogel exhibits a dual function, enhancing both angiogenesis and wound healing in vivo. Regarding tumor inhibition, the PEG@MT hydrogel demonstrated superior efficacy compared to intraperitoneal melatonin injection in vivo. In the liposarcoma resection niche, some tumor stem cells still survive, and can become activated, resulting in local and distant recurrence [45]. The systemic melatonin therapy faced challenges related to drug delivery and unstable blood concentrations, limiting its tumor inhibition effects [46]. However, the localized application of PEG@MT hydrogel enables the sustained release of melatonin directly at the local liposarcoma resection wound, effectively inhibiting residual tumor-stem cells and preventing liposarcoma recurrence.

## 3 Conclusion

In summary, we developed an injectable PEG@MT hydrogel with high adhesion and sustained release of melatonin to prevent local recurrence of liposarcoma and promote postoperative wound healing. To accommodate the high tension of the wound and conveniently operational application, the PEG@MT hydrogel is formed via amide bond covalently to achieve a high skin tension and can be easily injected with a syringe to cover the wound. In vitro studies confirmed that the PEG@MT hydrogel effectively promotes the fibroblast proliferation and angiogenesis. In addition, the PEG@MT hydrogel inhibits the tumor progression, and promotes cell cycle arrest and apoptosis via the FOXO signaling pathway. An in vivo tumor resection mouse model demonstrated the therapeutic efficiency of the PEG@MT hydrogel by significantly promoting wound healing, enhancing angiogenesis and inhibiting tumor proliferation and local recurrence in liposarcoma. Therefore, the use of injectable PEG@MT hydrogel with high adhesion for the postoperative tumor-resection wounds represents a valuable treatment strategy for patients with liposarcoma.

## CRediT authorship contribution statement

**Yonghui Liang:** Writing – original draft, Conceptualization. **Jianyang Shan:** Methodology, Conceptualization. **Chen Tan:** Validation, Investigation. **Zhen Pan:** Methodology, Funding acquisition. **Zhaohui Li:** Methodology, Investigation. **Xiang Fei:** Methodology, Investigation. **Gen Wen:** Supervision, Conceptualization. **Qingcheng Yang:** Funding acquisition, Conceptualization. **Dongdong Cheng:** Writing – review & editing, Supervision.

## Declaration of competing interest

The authors declare that they have no known competing financial interests or personal relationships that could have appeared to influence the work reported in this paper.

## Data Availability

Data will be made available on request.
