# Supplementary material for: A highly adhesive and melatonin-loaded PEG hydrogel prevents tumor recurrence and promotes wound healing for tumor-resection wound management of liposarcoma
Source: Mater Today Bio. 2025 May 6;32:101842. doi: 10.1016/j.mtbio.2025.101842 (PMC12136914; doi:10.1016/j.mtbio.2025.101842)
Supplement: Multimedia component 1 [file mmc1.docx]

Supporting Information


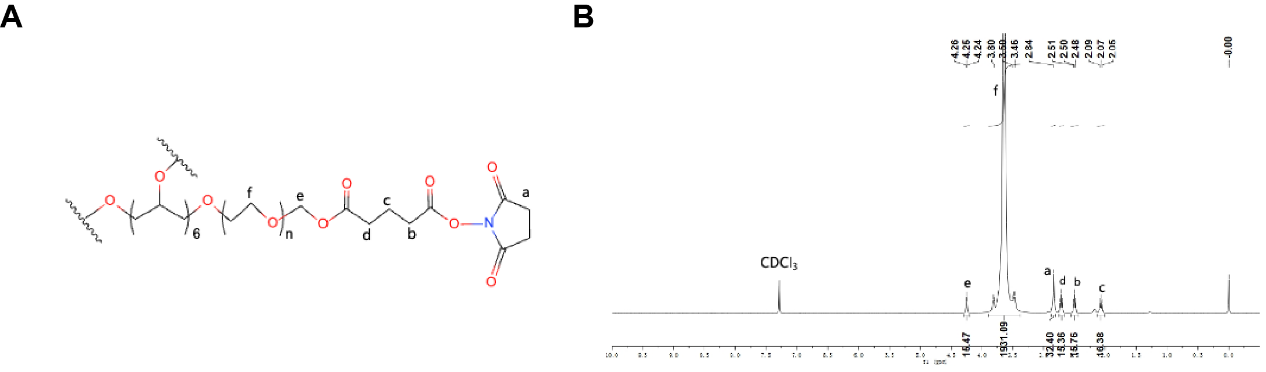


**Figure S1** (A)The molecular structure of 8-arm-PEG-SG. (B) 1H NMR spectra of 8-arm-PEG-SG.


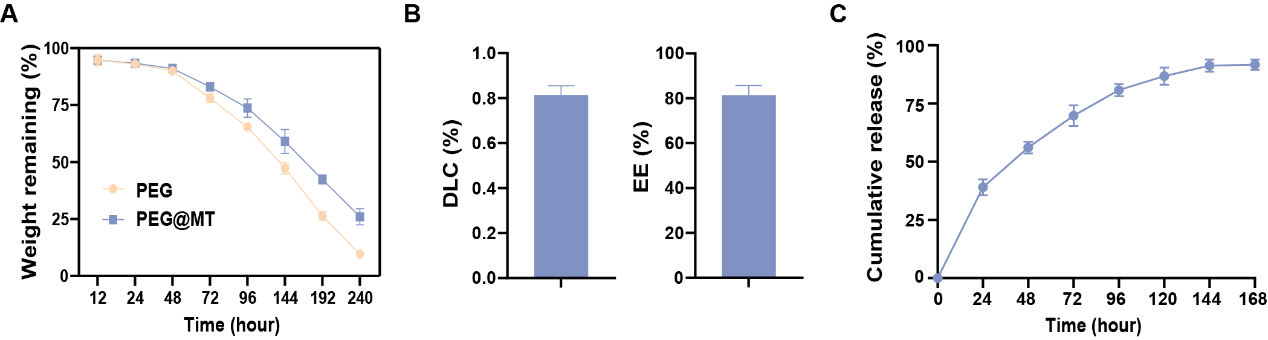


**Figure S2** (A)Time-dependent weight changes of PEG and PEG@MT hydrogels (n=3). (B) The Drug Loading Content (DLC%) and the Encapsulation Efficiency (EE%) of the PEG@MT hydrogel (n=3). (C) The release profiles of melatonin from PEG@MT hydrogels (n=3).


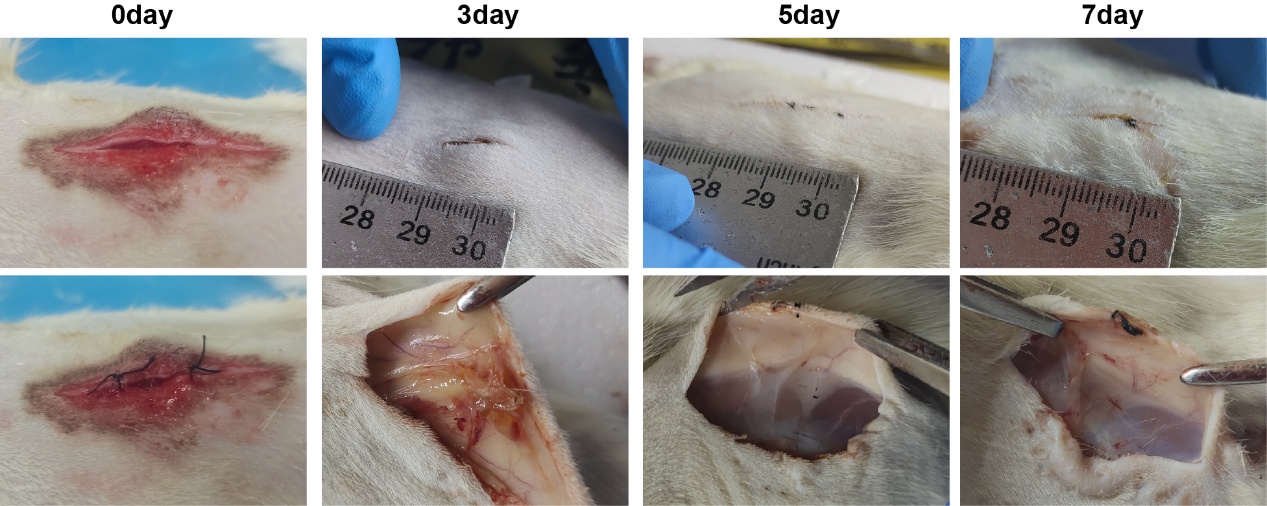


**Figure S3** The degradation of PEG@MT hydrogel under the dorsal side of the rats.


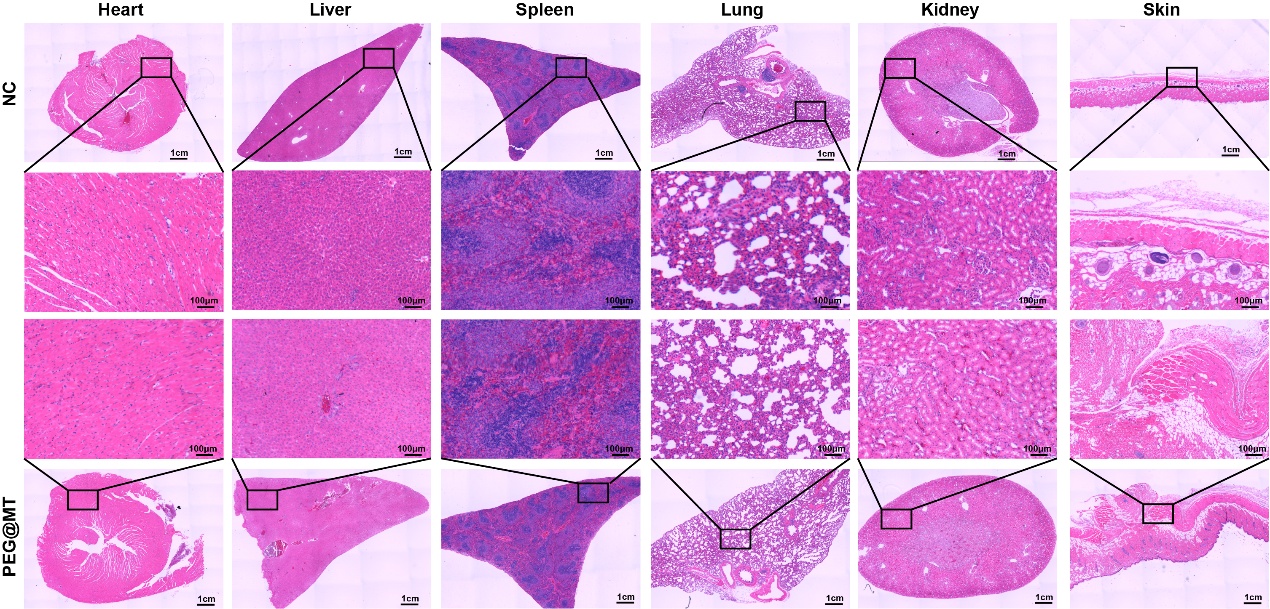


**Figure S4** H&E staining of heart, liver, spleen, lung, kidney, and skin tissues 7 days after injection of the PEG@MT hydrogel into the dorsal side of the rats.


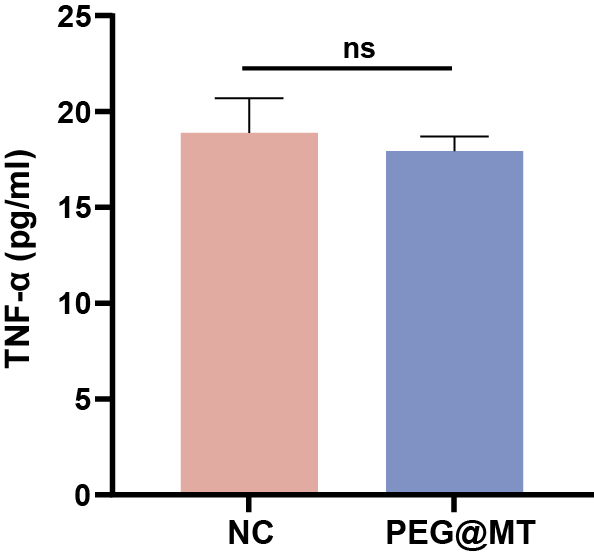


**Figure S5** The level of TNF-α in the serum of rats 7days after injection of the PEG@MT hydrogel (n=3).


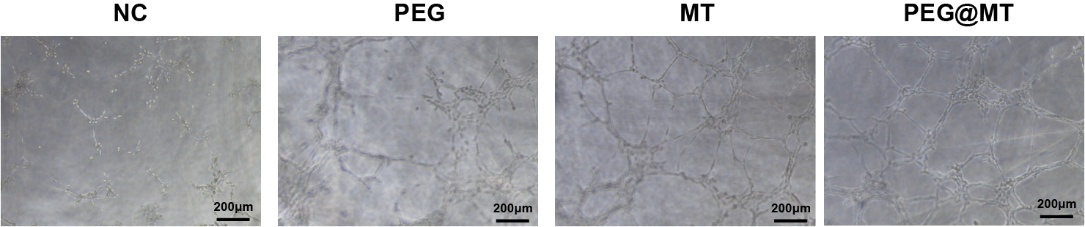


**Figure S6** The tube formation assay in HUVEC cells after incubation with the PEG hydrogel, melatonin, and PEG@MT hydrogel for 48 h (n=3).


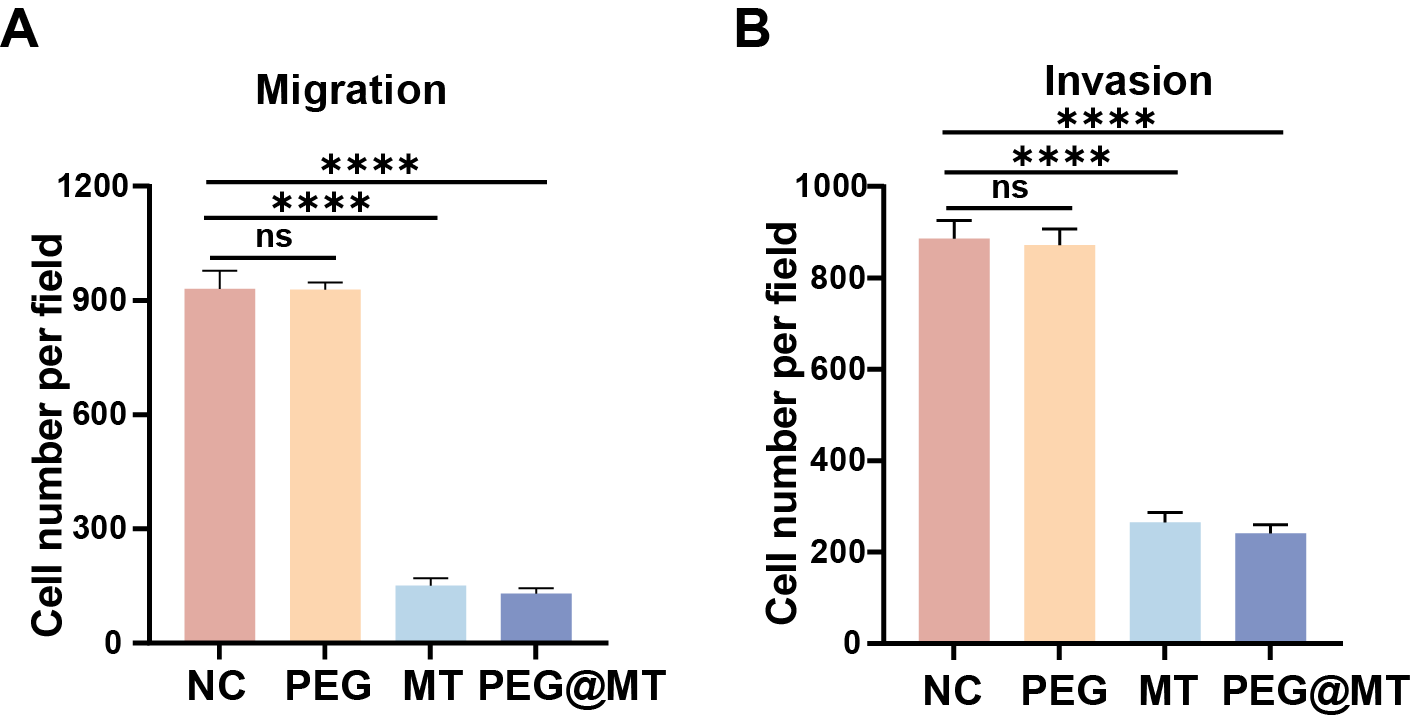


**Figure S7** The quantitative analysis of the migration and invasion capacities in SW872 cells after incubation with the PEG hydrogel, melatonin, and PEG@MT hydrogel for 48 h (n=3). ****p<0.0001.


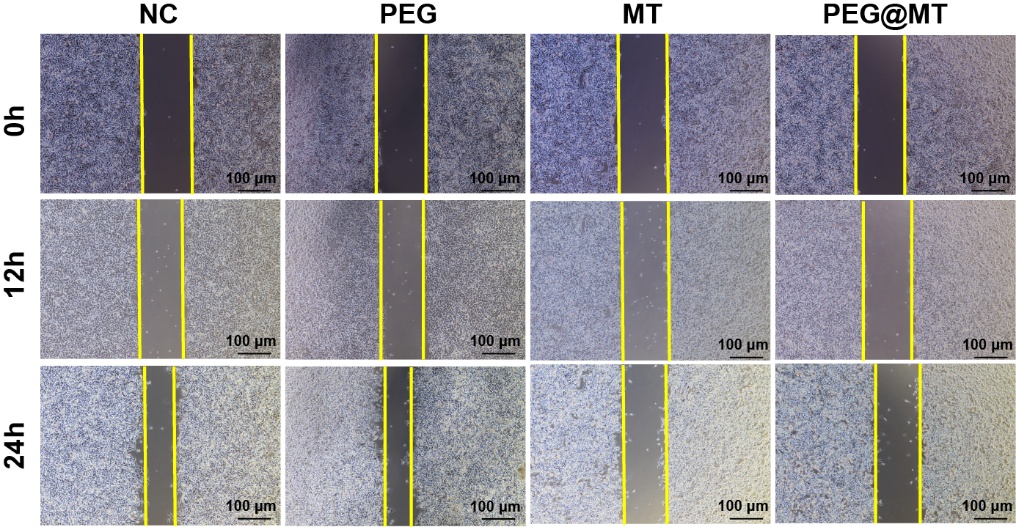


**Figure S8** The cell scratch experiment of SW872 cells after incubation with the PEG hydrogel, melatonin, and PEG@MT hydrogel for 12 h and 48 h (n=3).


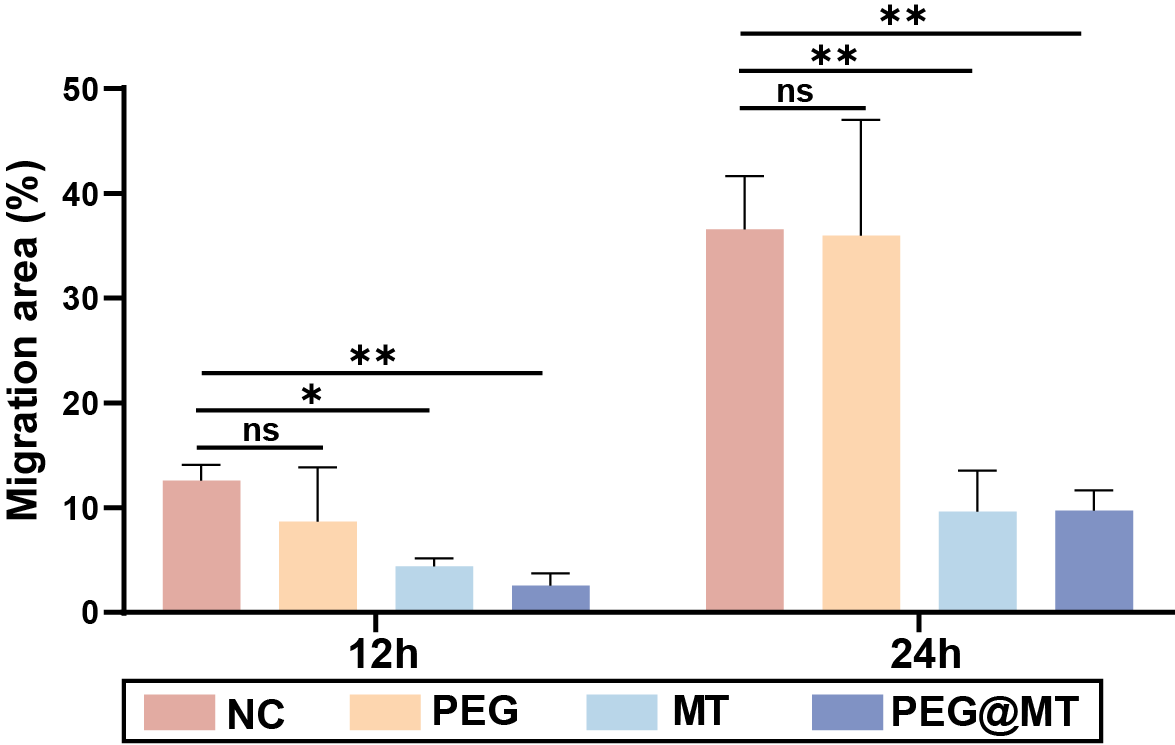


**Figure S9** The quantitative analysis of the cell scratch experiment of SW872 cells (n=3). **p<0.01; ***p<0.001.


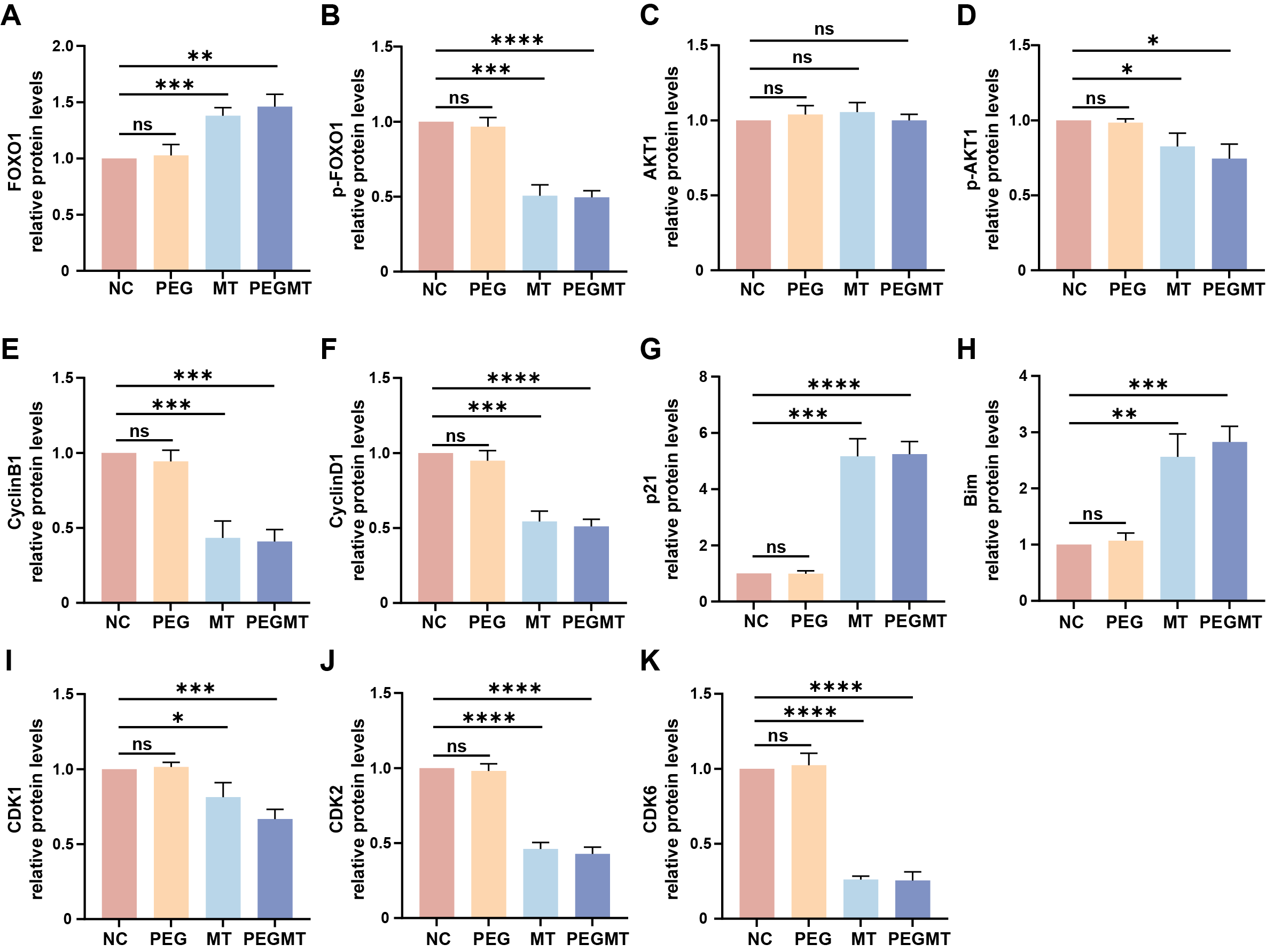


**Figure S10** The quantitative analysis of the expression of different proteins in Figure 5E (n=3). (A) FOXO1; (B) p-FOXO1; (C) AKT1; (D) p-AKT1; (E) CyclinB1; (F) CyclinD1; (G) p21; (H) Bim; (I) CDK1; (J) CDK2; (K) CDK6. *p<0.05; **p<0.01; ***p<0.001; ****p<0.0001.


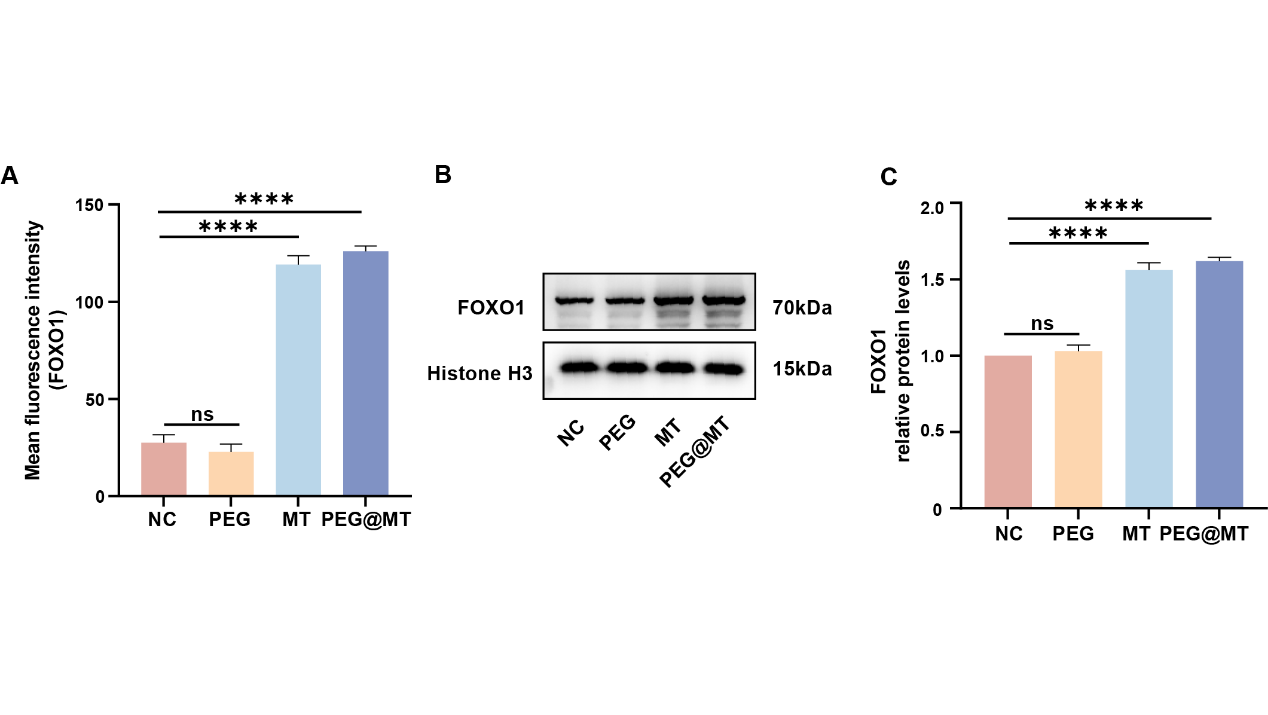


**Figure S11** (A) The quantitative analysis of the expression of FOXO1 in Figure 5F (n=3). (B-C) The expression of nuclear FOXO1 in SW872 cells with PEG hydrogel, MT and PEG@MT hydrogel treatment (n=3). ****p<0.0001.


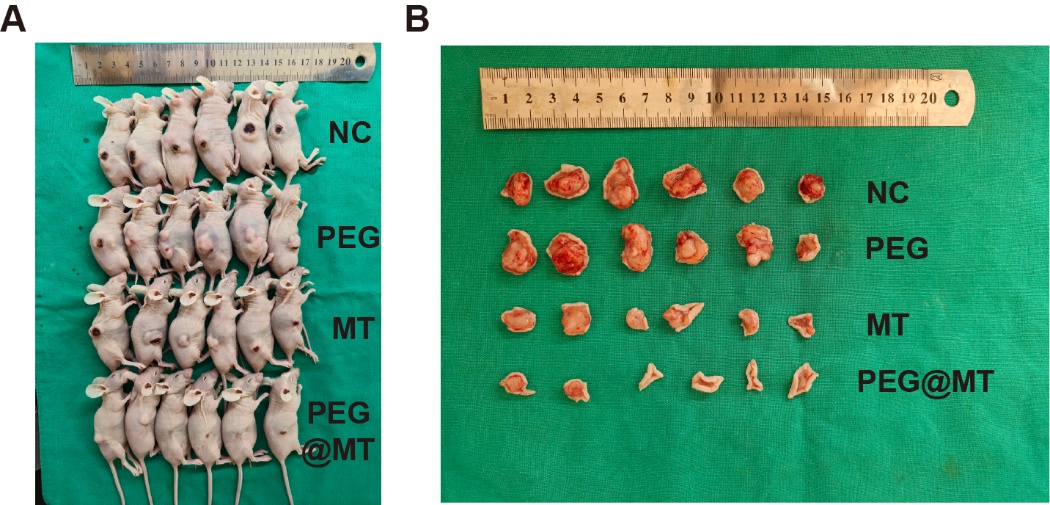


**Figure S12** (A) A photograph of tumor-bearing mice was shown (n=6). (B) A photograph of the tumors and the skins was shown when the mice were euthanized.


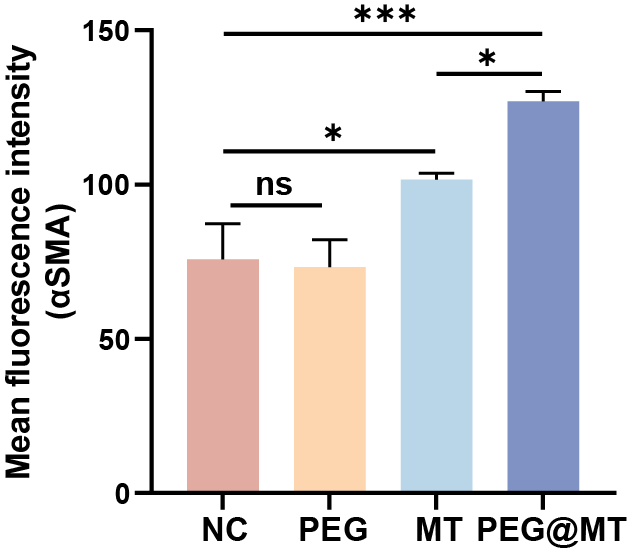


**Figure S13** The quantitative analysis of the expression of αSMA in Figure 7A (n=3). *p<0.05; ***p<0.001.


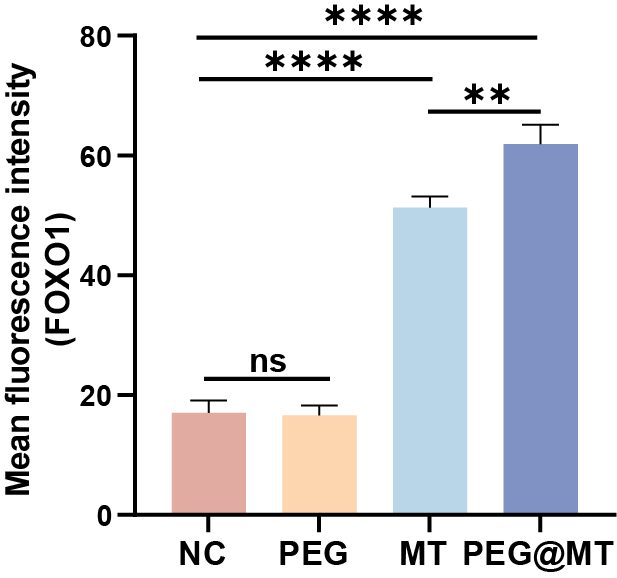


**Figure S14** The quantitative analysis of the expression of FOXO1 in Figure 7C (n=3). **p<0.01; ****p<0.0001.


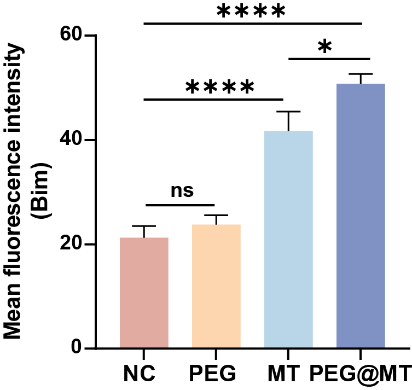


**Figure S15** The quantitative analysis of the expression of Bim in Figure 7C (n=3). *p<0.05; ****p<0.0001.
