# Supplementary material for: A highly adhesive and melatonin-loaded PEG hydrogel prevents tumor recurrence and promotes wound healing for tumor-resection wound management of liposarcoma
Source: Mater Today Bio. 2025 May 6;32:101842. doi: 10.1016/j.mtbio.2025.101842 (PMC12136914; doi:10.1016/j.mtbio.2025.101842)
Supplement: Multimedia component 2 [file mmc2.docx]

**1. Experimental Section**

*1.1. Materials:*

The 8-arm-PEG-SG and the 4-arm-PEG-NH_2_ were purchased from Xi’an QIYUE Biology (China). The melatonin was purchased from Selleck. The Annexin V-FITC Apoptosis Detection Kit, Crystal Violet Staining Solution, Hematoxylin and Eosin Staining Kit, Cell Cycle Analysis Kit and BeyoClick™ EdU Cell Proliferation Kit with Alexa Fluor 488 were purchased from Beyotime (China). The Reactive Oxygen Species Assay Kit and the Live-dead Assay were purchased from MeilunBio (China). The TNF-α ELISA Kit were purchased from Servicebio (China). The anti-FOXO1, anti-pFOXO1, anti-Akt1, anti-pAkt1 and anti-Bim were purchased from HUABIO (China). The anti-cyclinB1, anti-cyclinD1, anti-p21, anti-CDK1, anti-CDK2, anti-CDK6, and β-actin were purchased from Proteintech (China). The mouse L929 cell lines, the human SW872 cell lines and the Human umbilical vein endothelial cells (HUVECs) were purchased from Procell (China). All other reagents and materials were of high quality and used as received unless otherwise stated.

*1.2. Preparation of PEG and PEG@MT Hydrogels:*

The PEG@MT hydrogel was prepared by mixing the 8-arm-PEG-SG and the 4-arm-PEG-NH_2_ at a ratio of 1:1. Both their solid content were 5%. Concretely, 1mg melatonin and 25mg of 8-arm-PEG-SG were dissolved in 500 μL of PBS to obtain a clarified Solution of A, and 25mg of 4-arm-PEG-NH_2_ was dissolved in 500 μL of PBS to obtain a clarified Solution of B. Then, Solution B was added to Solution A, and the mixed solution turned into hydrogels at indoor temperature for a short time. For the PEG hydrogel, the melatonin was added in the Solution A. The formation of hydrogel was tested by a vial turnover test.

*1.3. The Rheological Measurements:*

Hydrogel samples (1 cm diameter × 1 cm height) were analyzed using a rotational rheometer. Time-sweep tests (1% strain, 1 Hz frequency) were performed to monitor storage modulus (G′) and loss modulus (G″) during gelation. Frequency sweeps (0.1–10 Hz, 1% strain) were conducted to assess viscoelastic properties. All measurements were carried out at 25°C.

*1.4. SEM:*

Freeze-dried hydrogels were sectioned, sputter-coated with gold, and imaged using scanning electron microscopy (SEM) at 10 kV accelerating voltage.

*1.5. Hydrogel adhesion capacity tests:*

Adhesive strength was quantified using a universal tensile tester. PEG@MT hydrogel (1 cm²) was sandwiched between two porcine skin strips. The force required to detach the hydrogel was measured at a 10 mm/min displacement rate. GelMA hydrogel served as a control.

*1.6. Hydrogel Degradability Assay:*

Pre-weighed freeze-dried hydrogels (W₀) were immersed in PBS (pH 7.4) at 37°C. At designated time points, samples were removed, freeze-dried again, and reweighed (Wₜ). Weight loss was calculated as: Weight loss (%) = (​*W*_0_​−*W_t_*_​​_) /*W*_0_×100%

*1.7. Drug loading into hydrogels*

The drug loading process was conducted according to the following procedure. Firstly, 25mg 8-arm-PEG-SG was dissolved in 500μL melatonin (1000 μg/mL), and were mixed with 500 μL 8-arm-PEG-SG solution, then the PEG@MT hydrogel was formed. The hydrogels were washed with PBS, freeze-dried, and then dried hydrogels were immersed in PBS to collect un-loaded melatonin. The supernatant was analyzed by UV–vis spectrophotometry (277 nm wavelength) to determine drug loading efficiency. The Encapsulation Efficiency (EE%) was calculated according to the following equation: EE (%)= (Actual MT loading / Initial MT loading)×100%. The Drug Loading Content (DLC%)=DLC (%)= (Extracted MT/ Dry hydrogel weight)×100%.

*1.8. Release Curve of Melatonin*

The melatonin-loaded hydrogel was located in 5 mL of PBS at 37 °C. The drug release was measured every day within 7 days, and the solution was measured by a UV–Vis spectrophotometer. The wavelength of melatonin was 277 nm.

*1.9. Cell Culture:*

Mouse L929 cell line, human SW872 liposarcoma cell line and human HUVEC cell line were bought from Procell (China). The L929 and SW872 cell lines were maintained in DMEM supplemented with 10% FBS and 1% penicillin/streptomycin. The HUVEC cell line were maintained in customized medium for HUVEC cells.

*1.10. Cytotoxic activity test:*

L929 cells were seeded at a density of 3000 cells well^−1^ in 96-well culture plates. After the cells had reached 70–80% confluence, the cells were treated with the leach liquor of PEG and PEG@MT hydrogel(leach liquor: complete medium ratio of 1:1), and the 2mM melatonin for different time. The PBS was added in the same ratio for control in the NC group. CCK-8 was added to each well and incubated for 2 h. The absorbance at 450 nm was measured using a microplate reader. Cell viability was calculated using the formula:

$$\frac{A_{s}- A_{b}}{A_{c}- A_{b}}\times100\%$$

where *As* is the absorbance of the sample, *Ac​* is the absorbance of the negative control sample, and *A_b_*​ is the absorbance of the blank sample.

*1.11. Live/Dead Staining:*

L929 cells were seeded at a density of 20000 cells well^−1^ in 24-well culture plates. After the cells had reached 70–80% confluence, the cells were treated with the leach liquor of PEG and PEG@MT hydrogel(leach liquor: complete medium ratio of 1:1) for 24 h. The cells were incubated by the staining kit for 30 min, and observed by an inverted fluorescence microscope. For the 3D incubation, the cell suspension was added to the solution B, and the hydrogel formed in the confocal dish. After being incubated by the staining kit for 30 min, the cells were observed by a laser confocal microscopy.

*1.12. CCK8 assay for tumor inhibition:*

The PEG and PEG@MT hydrogels formed in the 96-well culture plates, and the SW872 cells were seed at a density of 5000 cells well^−1^ and incubated for different time. Then CCK-8 was added to each well and incubated for 2 h. The absorbance at 450 nm was measured using a microplate reader.

*1.13. Clone formation assay:*

The PEG and PEG@MT hydrogels formed in the 6-well culture plates, and the SW872 cells were seed at a density of 15000 cells well^−1^ and incubated for 48 h. Then the cells were harvested and calculated. Then the cells were seed at a density of 1000 cells well^−1^. After 7 days, the cells were stained by the Crystal Violet Staining Solution and the number of colonies were calculated.

*1.14. Edu assay:*

The PEG and PEG@MT hydrogels formed in the 24-well culture plates, and the SW872 cells were seed at a density of 20000 cells well^−1^ and incubated for 48 h. Then the cells were staining by the BeyoClick™ EdU Cell Proliferation Kit with Alexa Fluor 488, and were observed by an inverted fluorescence microscope. The ratio of Edu-positive cells was calculated.

*1.15. Cell apoptosis and cell cycle analysis:*

The PEG and PEG@MT hydrogels formed in the 6-well culture plates, and the SW872 cells were seed at a density of 15000 cells well^−1^ and incubated for 48 h. After 48 h, cells were collected and measured via the Annexin V-FITC Apoptosis Detection Kit (Beyotime, China). The data were analyzed by FlowJo (USA). Cell cycle distribution was measured using a Cell Cycle Detection Kit (Beyotime, China), and was analysis by FlowJo (USA).

*1.16. Cell migration and invasion assay:*

Transwell chambers were used in the cell migration assay. The PEG and PEG@MT hydrogels formed in the 6-well culture plates, and the SW872 cells were seed at a density of 15000 cells well^−1^ and incubated for 48 h. Fifty thousand cells were harvested, calculated and added into the upper chamber per well. Meanwhile, 800 μl of DMEM containing 10% fetal bovine serum (FBS) was added into the lower chamber. After 24 h, the cells were fixed. After staining with crystal violet, the fixed cells were assessed under microscope.

*1.17. C**ell scratch experiment:*

L929 cells were seeded in 6-well culture plates for 48 h to form a cell monolayer. Subsequently, the cell monolayer was scratched using a 10 μL pipette and then washed twice with PBS. The cells were incubated with the PEG and PEG@MT hydrogel extracts, and cell images were captured at specific times using an inverted fluorescence microscope. Scratch healing rate(%)=(C_0_–C_t_)/C_0_×100%, where C_0_ and C_t_ represent the scratch area before and after intervention, respectively.

*1.18. Tube formation assay:*

Tube formation capacity was conducted using Matrigel. Matrigel (50 μL) was added to precooled 96-well plates and then placed at 37°C in a 5% CO^2^ ambience for 30 min. For angiogenesis assay of the melatonin, HUVECs (2×10^4^ cells/well) were incubated with 2mM melatonin for 48 h, then the cells were cultured in the plates. For angiogenesis assay of the hydrogels, HUVECs (2×10^4^ cells/well) were cultured in the hydrogel matrix. After incubating for 6 h, tube formation images were obtained by an inverted microscope, and tube numbers were calculated.

*1.19. ROS level test:*

L929 cells were seed at a density of 20000 cells well^−1^ in 24-well culture plates, and pre-treated with melatonin or hydrogel extracts for 48 h. Then, the cells were incubated by the Reactive Oxygen Species Assay Kit and observed by an inverted fluorescence microscope.

*1.20. Construction of RNA sequencing libraries and sequencing:*

Total RNA was extracted from the samples by Trizol reagent (Invitrogen) separately. The RNA quality was checked by Agilent 2200 and kept at −80°C. The RNA with RIN (RNA integrity number) > 7.0 is acceptable for cDNA library construction. The cDNA libraries were constructed for each RNA sample using the VAHTS Universal V6 RNA-seq Library Prep Kit for Illumina (Vazyme, Inc.) according to the manufacturer’s instructions. Generally, the protocol consists of the following steps: Poly-A containing mRNA was purified from 1ug total RNA using oligo(dT) magnetic beads and fragmented into 200-600 bp using divalent cations at 85℃ for 6 min. The cleaved RNA fragments were used for first- and second-strand complementary DNA (cDNA) synthesis. dUTP mix was used for second-strand cDNA synthesis, which allows for the removal of the second strand. The cDNA fragments were end repaired, A-tailed and ligated with indexed adapters. The ligated cDNA products were purified and treated with uracil DNA glycosylase to remove the second-strand cDNA. Purified first-strand cDNA was enriched by PCR to create the cDNA libraries. The libraries were quality controlled with Agilent 2200 and sequenced by DNBSEQ-T7 on a 150 bp paired-end run.

*1.21. RNA sequencing Mapping:*

Before read mapping, clean reads were obtained from the raw reads by removing the adaptor sequences and low-quality reads. The clean reads were then aligned to human genome (GRCh38, Ensembl104) using the star. HTseq was used to get gene counts and RPKM method was used to determine the gene expression.

*Western blotting:* cells were pre-treated with melatonin and hydrogel extracts for 48 h. The cells were then collected and lysed using RIPA buffer in an ice bath. The total content of the protein was detected according to the BCA method. Protein extracts were separated by SDS-PAGE and transferred to polyvinylidene fluoride membranes (Immobilon P, Millipore, Billerica, USA). Blots were blocked with 5% milk in Tris-buffered saline containing 0.1% Tween-20 for 1 h at room temperature. The membranes were incubated with primary antibodies at 4°C overnight, followed by incubation with the horseradish peroxidase-conjugated secondary antibodies at 37°C for 1 h. The immunoreactive bands were visualized by the Tanon Chemi Dog 5200T automatic luminescence imaging system.

*1.22. Immunofluorescent staining:*

SW872 cells were seed at a density of 20000 cells well^−1^ in 24-well culture plates. After culturing with the hydrogel extracts for 48 h, the cells were stained with the primary antibody and fluorescently labeled secondary antibody, and the nuclei was stained with DAPI. The cell immunofluorescence images were photographed with an inverted fluorescence microscope.

*Animal experiments:* All animal experiments were conducted in accordance with guidelines approved by the Institutional Animal Care and Use Committee (IACUC Issue NO: CX052502046).

*1.23. In vivo tumor recurrence evaluation:*

The back of SPF 4-week-old female nude mice were subcutaneously inoculated with SW872 cells (5.0×10^6^ cells per mouse) to establish melanoma tumor model. When the tumor volume reached 100 mm^3^, a circular full-thickness skin defect wound (8 mm) was constructed at the tumor site and ~90% tumor tissue was removed. Then the mice were randomly divided into 4 groups: (1) Control; (2) PEG hydrogel; (3) MT; (4) PEG@MT hydrogel. In the PEG group, PEG hydrogel was injected and formed a covering over the postoperative wound. The MT group was conducted by intraperitoneal injection with 200 μL of a solution of melatonin (100 mg/kg, every 2 days). In the PEG@MT group, 5mg melatonin was embedded in 1 cm^3^ PEG hydrogel, and the hydrogel was injected and formed a covering over the postoperative wound. The body weight and tumor volume of mice were recorded every 2 days within 8 days. The tumor volume (mm^3^) was calculated as (tumor width)^2^×(tumor length)×0.52. The wounds on the back of the nude mice were photographed every 2 days. Animals were sacrificed after 8 days, and the tumors were weighed. The tumor and skin tissue were used for H&E staining, TUNEL staining and Immunofluorescent staining.

*1.24. In Vivo Toxicity assessment:*

The acute toxicity of PEG@MT hydrogels was determined in rats. After shaving the back of rats, 2 cm incision was made in the back. 1ml of the hydrogels was subcutaneously injected into the back of rats by a needle. The rats were sacrificed after 3, 5 and 7 days, and the hydrogels were photographed. After 7 days, the rats were euthanized and the main organs including liver, kidney, spleen, and the skin at the site of injection were collected and fixed in 4% formaldehyde for 8 h, followed by embedding in paraffin and sectioning by microtome to slides before H&E staining.

*1.25. Statistical Analysis:*

Statistical analyses were performed using GraphPad Prism 9 software. All data are presented as the mean ± SD (n = 3). When comparing only two groups, statistical signiﬁcance was determined using the student’s t-test. When comparing more than 2 group, One-Way ANOVA and Turkey’s multiple comparison test were conducted. Statistical signiﬁcance was deﬁned as *p < 0.05, **p < 0.01, ***p < 0.001 and ****p < 0.0001.
